# Phase-Related Resting Energy Expenditure in Critically Ill Adults: Metabolic Phenotypes and Determinants of Weight-Normalized Indices—A Retrospective Study

**DOI:** 10.3390/jcm15010237

**Published:** 2025-12-28

**Authors:** Sebastián Chapela, Jaen Cagua-Ordoñez, Jaime Angamarca-Iguago, Daniel Tettamanti, Claudia Kecskes, Jesica Asparch, Facundo Javier Gutierrez, Natalia Llobera, Mariana Rella, Martha Montalván, María Jimena Reberendo, Mario Omar Pozo, Ludwig Álvarez-Córdova, Daniel Simancas-Racines

**Affiliations:** 1 Departamento de Bioquímica, Facultad de Medicina, Universidad de Buenos Aires, Ciudad Autónoma de Buenos Aires C1121ABG, Argentina; sebachapela@gmail.com; 2 Unidad de Soporte Nutricional, Hospital Británico de Buenos Aires, Ciudad Autónoma de Buenos Aires C1280AEVB, Argentina; naty.llobera@gmail.com (N.L.); jimereberendo@gmail.com (M.J.R.); 3 Escuela de Medicina, Pontificia Universidad Católica del Ecuador, Santo Domingo 230203, Ecuador; jccagua@pucesd.edu.ec (J.C.-O.); jwangamarca@pucesd.edu.ec (J.A.-I.); 4 Facultad de Ciencias de la Salud y Bienestar Humano, Universidad Tecnológica Indoamérica, Quito 170103, Ecuador; 5 Escuela de Medicina, Universidad Católica de Santiago de Guayaquil, Av. Pdte. Carlos Julio Arosemena Tola, Guayaquil 090615, Ecuador; dantettamanti@gmail.com; 6 Sección de Soporte Nutricional, Hospital Italiano, Ciudad Autónoma de Buenos Aires C1439BSN, Argentina; claudia.kecskes@hospitalitaliano.org.ar (C.K.); jesica.asparch@gmail.com (J.A.); 7 Universidad del Hospital Italiano, Ciudad Autónoma de Buenos Aires C1439BSN, Argentina; 8 Servicio de Terapia Intensiva, Hospital Británico de Buenos Aires, Ciudad Autónoma de Buenos Aires C1280AEVB, Argentina; facundo_gutierrez@yahoo.com.ar (F.J.G.); pozomario@hotmail.com (M.O.P.); 9 Servicio de Endocrinología, Nutrición y Diabetes, Hospital Británico de Buenos Aires C1280AEVB, Argentina; marianarella2024@gmail.com; 10 Escuela de Medicina, Universidad Espíritu Santo, Samborondón 0901952, Ecuador; mmontalvanmd53@gmail.com; 11 Maestría de Nutrición y Dietética, Facultad de Ciencias de la Salud, Universidad de Las Américas (UDLA), Quito 170124, Ecuador; 12 Facultad de Salud y Bienestar, Pontificia Universidad Católica del Ecuador, Quito 170143, Ecuador; dasimancas@puce.edu.ec; 13 Facultad de Ciencias de la Salud y Bienestar Humano, Universidad Tecnológica Indoamérica, Ambato 180150, Ecuador

**Keywords:** indirect calorimetry, resting energy expenditure, critical illness, metabolic phenotype, mechanical ventilation, nutritional support

## Abstract

**Background:** Precise measurement of resting energy expenditure (REE) is essential in the intensive care unit (ICU), where metabolic requirements evolve throughout the course of critical illness. Predictive equations frequently fail to capture this variability, and limited data describe phase-dependent changes in REE using indirect calorimetry (IC). This study aimed to evaluate phase-related variation in REE and metabolic phenotypes in mechanically ventilated adults and to identify clinical and physiological correlates of both absolute REE and REE normalized by ideal body weight (REE/IBW). **Methods:** We conducted an observational, retrospective cross-sectional study in two ICUs at different hospitals. A total of 149 mechanically ventilated adults with a valid IC measurement were included and classified by illness phase: acute (0–3 days), intermediate (4–14 days), or chronic (>14 days). Differences in metabolic and gas-exchange variables were assessed using ANOVA or Kruskal–Wallis tests. Two multivariable linear regression models were fitted, one using absolute REE and a second using REE/IBW, incorporating metabolic phenotype categories to account for body-size heterogeneity. **Results:** Metabolic profiles differed across illness phases. Median REE increased from the acute (1664 kcal/day) to the intermediate (1869 kcal/day) and chronic (2074 kcal/day; *p* = 0.024) phases. Hypometabolic profiles were more frequent in the acute phase (64%), whereas hypermetabolic profiles were more prevalent in later phases (48%). RQ values were higher in the chronic phase compared with the acute phase (median 0.99 vs. 0.80; *p* < 0.001). In multivariable analyses, illness severity scores showed weak or inconsistent associations with REE after adjustment for gas-exchange variables. VCO_2_ was independently associated with absolute REE (adjusted R^2^ = 0.83). In the REE/IBW model, VCO_2_, RQ, BMI, and metabolic phenotype were associated with normalized energy expenditure, with higher adjusted R^2^ (0.87) and lower prediction error metrics. **Conclusions:** Resting energy expenditure and metabolic phenotypes vary across phases of critical illness. Gas-exchange variables, particularly VCO_2_, were more closely associated with measured energy expenditure than severity scores. Normalization of REE by ideal body weight reduced variability and improved model performance, highlighting the analytical value of indirect calorimetry for characterizing phase-dependent metabolic patterns in critically ill adults.

## 1. Introduction

Accurate measurement of energy expenditure in critically ill patients remains a key part of personalized nutrition therapy in the intensive care unit (ICU), where metabolic demands are both increased and highly variable throughout illness [1]. In contemporary critical care nutrition, the trajectory of critical illness is commonly conceptualized into phases (e.g., early acute, late acute/stabilization, and recovery/chronic), during which metabolic demands and the balance between endogenous and exogenous energy provision evolve [2,3,4].

Critical illness usually progresses from an initial, often energy-limited, phase into a sustained hypermetabolic state caused by inflammation, organ failure, and medical treatments. This progression raises concerns about the effectiveness of traditional predictive equations based on physiologically stable groups [5]. Moreover, early full feeding may increase the risk of overfeeding, because substantial endogenous energy production persists during the first days of acute illness [4].

Indirect calorimetry (IC) is the reference standard for bedside assessment of resting energy expenditure (REE), estimating metabolic rate through measurement of gas exchange and providing insight into substrate utilization and bioenergetic efficiency [6]. International guidance recommends IC for determining energy expenditure in mechanically ventilated critically ill adults when available, given the limitations of equation-based estimation in this population. Despite its clinical advantages, routine implementation is limited by operational barriers, leading many ICUs to rely on predictive equations such as the Harris–Benedict, Mifflin–St Jeor, or Penn State formulas that frequently misestimate energy requirements in mechanically ventilated patients with evolving physiology [7,8].

Studies comparing equations against IC have repeatedly demonstrated poor accuracy in mechanically ventilated ICU cohorts, reinforcing the risk of systematic under- or overestimation at the individual level [8]. These inaccuracies may contribute to clinically meaningful complications, including overfeeding-related metabolic stress or energy deficits that exacerbate muscle wasting and prolong ventilatory dependence [5,9]. These inaccuracies are magnified in patients with obesity, sepsis, multi-organ support (ECMO/CRRT), or protracted mechanical ventilation, where inter-individual metabolic variability is greatest [6,8].

High-quality evidence supports the clinical benefits of IC-guided nutrition. A recent systematic review and meta-analysis of randomized trials demonstrated reduced short-term mortality when caloric prescriptions were based on IC rather than predictive equations [10]. Updated syntheses continue to suggest potential short-term outcome benefits without an excess of adverse events, particularly in mechanically ventilated patients and when IC is reassessed over time [11]. International societies (ESPEN, ASPEN, SCCM) therefore endorse IC as the preferred method for determining energy requirements in critical illness, emphasizing phase-appropriate targets to avoid both under- and overfeeding [4]. In parallel, the respiratory quotient (RQ) provides complementary information regarding substrate oxidation, and values approaching or exceeding 1.0 may indicate predominant carbohydrate oxidation and/or potential overfeeding, supporting its clinical usefulness during nutrition titration [5,12]. Emerging evidence also suggests that metabolic phenotypes derived from gas-exchange patterns may carry prognostic implications [2].

Given these considerations, the objective of this study was to characterize phase-dependent variation in resting energy expenditure among mechanically ventilated critically ill adults and to quantify its determinants using indirect calorimetry. Specifically, we examined both absolute REE and REE normalized by ideal body weight (REE/IBW), evaluating their relationships with gas-exchange parameters (VCO_2_, RQ), clinical characteristics, and metabolic phenotypes to inform precision energy prescriptions across the trajectory of critical illness. Normalization by ideal body weight was selected to improve physiological comparability across a population with wide variability in body size and adiposity and to reduce scale-dependent distortion when interpreting weight-indexed energy expenditure [8,13].

Despite increasing evidence of metabolic variability in critical illness, to our knowledge, few studies have examined absolute and normalized REE, along with gas-exchange determinants and metabolic phenotype, across all phases of critical illness using indirect calorimetry. Recent large-cohort phase-based analyses further underscore that measured energy expenditure changes across early acute, late acute, and recovery phases, supporting the need for phase-aware assessment rather than a single static estimate [2,14].

## 2. Methods

### 2.1. Study Design and Setting

This was an observational, retrospective, cross-sectional comparative study conducted in two high-complexity intensive care units from two different tertiary-care hospitals. Each patient contributed a single valid indirect calorimetry (IC) measurement, classified according to the phase of critical illness at the time of assessment: acute (0–3 days), intermediate or stabilization phase (4–14 days), or chronic critical phase (>14 days). Because each record corresponded to a distinct patient, and no repeated IC measurements were available, longitudinal or mixed-effects modeling was not applicable.

### 2.2. Population and Eligibility Criteria

Eligible participants were adults (≥18 years) receiving invasive mechanical ventilation who underwent at least one complete and physiologically valid IC measurement under standardized conditions. Patients were identified through institutional IC registries and electronic medical records at both hospitals.

Exclusion criteria included recent tracheostomy (<48 h), excessive ventilatory circuit leaks (>10%), hemodynamic or ventilatory instability during measurement, recent procedures or enteral/parenteral boluses within the preceding 2 h, and incomplete clinical or metabolic data. Incomplete data were defined as missing IC-derived variables (VO_2_, VCO_2_, or RQ) or absence of key covariates required for multivariable analysis. Only patients with reliable IC measurements and complete covariate information were included.

### 2.3. Data Sources and Measurement Procedures

Clinical variables were extracted from electronic medical records, and metabolic data were obtained from IC device logs at each institution. Indirect calorimetry was performed using calibrated, ventilator-integrated metabolic monitors in accordance with manufacturer specifications and ICALIC methodological standards. Different device models were used across hospitals; standardized measurement protocols and daily calibration procedures were applied at both sites.

Each IC session lasted 20 to 30 min, with the final 10 to 15 min analyzed under steady-state conditions (variability < 10% for VO_2_ and VCO_2_ and <5% for the respiratory quotient). Recorded parameters included VO_2_, VCO_2_, RQ, and REE, calculated using the Weir equation. REE normalized by ideal body weight (REE/IBW) was also calculated using standard sex- and height-based formulas. Information on ventilator settings, sedation, vasoactive support, and nutritional delivery at the time of IC measurement was not available in a standardized manner across both sites.

### 2.4. Variables and Operational Definitions

The primary outcome variable was resting energy expenditure (REE, kcal/day) measured by indirect calorimetry. Body size-adjusted indices were derived as REE per actual body weight (REE/Weight) and REE per ideal body weight (REE/IBW). REE normalized by ideal body weight (REE/IBW) was pre-specified as a key derived index for subsequent modeling. Ideal body weight (IBW) was calculated using the Miller formula.

Metabolic parameters obtained from indirect calorimetry included oxygen consumption (VO_2_, mL/min), carbon dioxide production (VCO_2_, mL/min), and the respiratory quotient (RQ = VCO_2_/VO_2_). To facilitate size-adjusted comparisons of gas exchange, additional normalized indices were computed: VO_2_/weight, VO_2_/IBW, VCO_2_/weight, and VCO_2_/IBW. These variables were analyzed descriptively and in bivariate analyses only, to characterize phase-dependent variation in bioenergetic activity.

Clinical and demographic variables included age (years), weight (kg), height (cm), and body mass index (BMI, kg/m^2^). Severity indicators assessed at the time of indirect calorimetry comprised the Sequential Organ Failure Assessment (SOFA), Acute Physiology and Chronic Health Evaluation II (APACHE II), and Charlson Comorbidity Index (CCI). The phase of critical illness—acute phase (0–3 days), intermediate or stabilization phase (4–14 days), or chronic critical phase (>14 days)—was defined a priori and treated as the principal exposure variable for stratified analyses.

All quantitative variables were screened for physiological plausibility using predefined metabolic ranges (VO_2_: 100–500 mL/min; VCO_2_: 80–400 mL/min; RQ: 0.65–1.20). Values outside these ranges were systematically verified against source documentation, and unresolved discrepancies were treated as missing data.

### 2.5. Metabolic Categorization

A categorical metabolic phenotype variable was constructed to contextualize individual metabolic status relative to predicted energy expenditure. The percentage of predicted REE was calculated as

$$\%REE_{\mathrm{pred}}=\left(\frac{REE_{\mathrm{measured}}}{REE_{\mathrm{predicted}}}\right)\times 100$$

Based on commonly used clinical thresholds, patients were classified as hypometabolic (≤90% of predicted), normometabolic (90–110% of predicted), or hypermetabolic (≥110% of predicted) [15].

Predicted REE values were obtained using the Penn State 2003b equation, which was the only predictive formula consistently available across both study sites for mechanically ventilated critically ill adults. Predicted REE was used exclusively to derive the percentage-of-predicted REE metric and to define metabolic phenotype categories and was not used for any direct comparison with measured REE.

To prevent statistical endogeneity and mathematical coupling, predicted REE was not included as an explanatory variable in any multivariable model with measured REE as the dependent variable. The derived metabolic phenotype variable was incorporated as an independent covariate only in models evaluating REE normalized by ideal body weight (REE/IBW), and was not included in models of absolute REE.

### 2.6. Data Preparation and Transformation

All continuous variables were initially explored graphically using histograms, kernel density plots, and Q–Q plots to assess distributional properties and potential departures from normality. Exploratory assessments were performed on the pooled dataset, and potential site-related variability was evaluated subsequently as part of sensitivity analyses. Resting energy expenditure (REE) exhibited mild right-skewness; therefore, a log-transformed specification [log(REE)] was examined in sensitivity analyses. Because log-transformed models produced similar effect estimates and did not materially improve residual structure, all primary results were retained on the original scale (kcal/day) to preserve clinical interpretability.

Derived gas-exchange indices (VO_2_/weight, VO_2_/IBW, VCO_2_/weight, VCO_2_/IBW) were evaluated for distributional symmetry, physiological plausibility, and linearity relative to REE. Only absolute VO_2_ and VCO_2_ were retained as primary gas-exchange predictors for multivariable modeling to minimize collinearity, while normalized indices were used exclusively for descriptive and bivariate analyses. The respiratory quotient (RQ) was included only in the secondary normalized model (REE/IBW) to explore substrate-utilization patterns, acknowledging its correlation with VO_2_ and VCO_2_.

Potential outliers and influential observations were identified using standard diagnostic thresholds, including studentized residuals (|r| > 3), leverage values exceeding 2 k/n, and Cook’s distances greater than 4/n. All flagged observations had previously met predefined physiological plausibility criteria, and each was individually reviewed to verify measurement validity. Since none of the flagged cases materially influenced model coefficients or statistical inference in sensitivity analyses, all physiologically plausible observations were retained in the final models to preserve underlying population variability.

All variables were complete in the final analytical cohort, as patients with incomplete clinical or metabolic data were excluded during the eligibility screening process. Therefore, no missing data handling or imputation procedures were required for multivariable analyses.

### 2.7. Statistical Analysis

#### 2.7.1. Descriptive Analysis

Continuous variables were summarized as mean ± standard deviation (SD) or median with interquartile range (IQR), depending on their distribution, as assessed by Shapiro–Wilk tests and visual inspection of Q–Q plots. Categorical variables were described as absolute and relative frequencies. Metabolic phenotypes (hypometabolic, normometabolic, hypermetabolic) were tabulated across illness phases. All baseline, clinical, and metabolic variables were summarized descriptively, stratified by phase of critical illness, and presented in comprehensive tables.

#### 2.7.2. Comparative Analysis Between Phases

Differences across illness phases were assessed using one-way analysis of variance (ANOVA) for normally distributed variables with homoscedasticity verified by Levene’s test. When assumptions were not met, the Kruskal–Wallis test was applied. For statistically significant global tests, pairwise comparisons were performed using Tukey’s HSD (ANOVA) or Dunn’s test with Bonferroni correction (Kruskal–Wallis). Categorical variables were compared using Pearson’s χ^2^ test, or Fisher’s exact test where applicable (expected cell count < 5). In addition to *p*-values, effect sizes were reported (partial η^2^ for parametric tests and epsilon-squared [ε^2^] for nonparametric analyses) to quantify the magnitude and direction of between-phase differences. The association between illness phase and metabolic phenotype was examined using χ^2^ testing, as a descriptive assessment of distributional differences across phases, without implying validation between continuous and categorical representations of metabolism.

#### 2.7.3. Bivariate Correlation Analysis

Associations between REE and clinical or metabolic variables were evaluated using Pearson correlation coefficients for normally distributed variables and Spearman rank correlation coefficients (ρ) for skewed distributions or ordinal parameters. All correlations were presented with 95% confidence intervals and *p*-values. These analyses were intended to describe the strength and direction of pairwise associations and were not interpreted as independent effects.

Given the relevance of gas-exchange physiology in determining energy expenditure, correlations were additionally assessed for body size–adjusted indices (VO_2_/weight, VO_2_/IBW, VCO_2_/weight, VCO_2_/IBW). These exploratory analyses were conducted to characterize proportional and physiologically scaled relationships between gas-exchange parameters, substrate utilization (RQ), and REE, without inferring causality or independence.

#### 2.7.4. Multivariable Modeling

To identify independent predictors of resting energy expenditure (REE), two multivariable linear regression models were specified in accordance with the prespecified analytical framework.

Primary model (Model 1: Determinants of measured REE)

The primary model evaluated associations between clinical variables and directly measured REE (kcal/day). Covariates were selected a priori based on clinical relevance and prior evidence, with bivariate associations (*p* < 0.20) used only as a complementary screening criterion. The model included phase of critical illness (acute, intermediate, chronic), body mass index (BMI), Sequential Organ Failure Assessment (SOFA) score, Acute Physiology and Chronic Health Evaluation II (APACHE II) score, Charlson Comorbidity Index (CCI), and carbon dioxide production (VCO_2_). VO_2_ was not included, because of its strong correlation with VCO_2_ and to reduce collinearity in the multivariable setting.

Phase of illness was modeled as a categorical variable, with the acute phase as the reference category. SOFA and APACHE II scores were included concurrently as complementary indicators of illness severity, and model diagnostics were used to verify acceptable collinearity and coefficient stability. The ICU site was not included in the primary model and was examined separately in sensitivity analyses.

The model equation was expressed as

$$REE_{i}\;=\;\beta_{0}\;+\;\beta_{1}\;{\left(\mathrm{Phase}\right)}_{i}\;+\;\beta_{2}\;{\left(\mathrm{BMI}\right)}_{i}\;+\;\beta_{3}\;{\left(\mathrm{SOFA}\right)}_{i}\;+\;\beta_{4}\;{\left(\mathrm{APACHEII}\right)}_{i}\;+\;\beta_{5}\;{\left(\mathrm{CCI}\right)}_{i}\;+\;\beta_{6}\;{\left(\mathrm{VC}O_{2}\right)}_{i}\;+\;\varepsilon_{i}$$

where $REE_{i}$ is the measured resting energy expenditure for subject *i*, $\beta_{0}$ is the intercept, $\beta_{k}$ are partial regression coefficients, and $\varepsilon_{i}$ represents the random error term.

Secondary model (Model 2: Normalized REE with metabolic phenotype)

To avoid potential endogeneity between measured REE and its derived metabolic classification, a secondary model was constructed using REE normalized by ideal body weight (REE/IBW) as the dependent variable. This model assessed the association between metabolic phenotype and normalized energy expenditure, while adjusting for clinical covariates.

The model included phase of illness (categorical), BMI, SOFA score, respiratory quotient (RQ), VCO_2_, and metabolic phenotype (hypometabolic, normometabolic, hypermetabolic). Metabolic phenotype was treated as a three-level categorical variable, with normometabolic status as the reference category. Predicted REE was not included as an explanatory variable in any model.

The model equation was

$${\left(\frac{REE}{IBW}\right)}_{i}=\beta_{0}+\beta_{1}{\left(\mathrm{Phase}\right)}_{i}+\beta_{2}{\left(\mathrm{BMI}\right)}_{i}+\beta_{3}{\left(\mathrm{SOFA}\right)}_{i}+\beta_{4}{\left(\mathrm{RQ}\right)}_{i}+\beta_{5}{\left(\mathrm{VC}O_{2}\right)}_{i}+\beta_{6}{\left(\mathrm{MetabolicCategory}\right)}_{i}+\varepsilon_{i}$$

This formulation incorporated metabolic phenotype as an independent categorical covariate and avoided mathematical coupling with the dependent variable. As in the primary model, ICU site was evaluated in sensitivity analyses rather than included in the main specification.

#### 2.7.5. Model Diagnostics and Assumption Testing

Both multivariable models underwent a comprehensive diagnostic assessment to evaluate the adequacy of model assumptions and the robustness of statistical inference. Linearity between each continuous predictor and the dependent variable (REE or REE/IBW) was assessed using component-plus-residual and partial residual plots. When visual inspection suggested potential departures from linearity, restricted cubic splines with three knots (10th, 50th, and 90th percentiles) were prespecified and examined in sensitivity analyses to evaluate whether nonlinear specifications improved model fit.

Multicollinearity was examined using variance inflation factors (VIFs), with values below commonly accepted thresholds indicating acceptable levels of collinearity. Residual normality was assessed using Shapiro–Wilk tests and Q–Q plots, whereas homoscedasticity was evaluated using the Breusch–Pagan and White tests. When evidence of heteroscedasticity was detected, heteroskedasticity-consistent standard errors (HC3) were applied to ensure robust variance estimation.

Residual independence was assessed using the Durbin–Watson statistic. Given that each participant contributed a single indirect calorimetry measurement, serial correlation was not anticipated; nevertheless, this diagnostic was performed as part of routine model verification.

Influential observations were identified using leverage values, standardized residuals, and Cook’s distance, based on conventional diagnostic thresholds (h > 2 k/n, |r| > 3, Cook’s D > 4/n). Flagged observations were reviewed for physiological plausibility and data integrity, and sensitivity analyses were conducted by refitting models after excluding influential cases to assess the stability of parameter estimates.

Model adequacy and parsimony were evaluated using adjusted R^2^, Akaike Information Criterion (AIC), the small-sample–corrected AIC (AICc), and the Bayesian Information Criterion (BIC). Predictive performance was quantified using root mean square error (RMSE) and mean absolute error (MAE). Internal model stability was assessed through 10-fold cross-validation with a fixed random seed, with models iteratively trained on nine folds and evaluated on the remaining fold to obtain out-of-sample RMSE and MAE estimates.

Because resting energy expenditure is calculated using both VO_2_ and VCO_2_ through the Weir equation, potential mathematical coupling between gas-exchange variables and the outcome was explicitly explored in prespecified sensitivity analyses. Alternative model specifications excluding VCO_2_, models including VO_2_ alone, and spline-based formulations were examined to evaluate the consistency of associations across specifications.

In addition, to address potential center-related heterogeneity arising from data collection in two independent intensive care units, sensitivity analyses were performed incorporating ICU site as an additional covariate and by refitting models stratified by ICU. These analyses were conducted to assess whether center-level differences in clinical practice, measurement context, or case mix materially influenced model estimates. Across sensitivity analyses, the direction and relative magnitude of associations were examined for consistency, and these analyses were intended to evaluate the robustness of findings rather than to establish causal independence.

#### 2.7.6. Statistical Threshold and Software

All regression coefficients (β) were reported with their corresponding 95% confidence intervals and two-tailed *p*-values, with *p* < 0.05 considered indicative of statistical significance. Statistical inference was interpreted in conjunction with effect sizes and model performance metrics, as described above. Analyses were performed using R software (version 4.5). The following packages were used: stats, tidyverse, car, lmtest, sandwich, broom, AICcmodavg, gtsummary, and splines.

#### 2.7.7. Ethical Considerations

The study was conducted in accordance with the principles of the Declaration of Helsinki and applicable institutional research ethics standards. The protocol was reviewed and approved by the corresponding institutional ethics committee. Given the retrospective nature of the study and the exclusive use of anonymized data without direct patient contact, the requirement for informed consent was waived.

## 3. Results

### 3.1. Study Population and Demographic Characteristics

**Figure 1:**
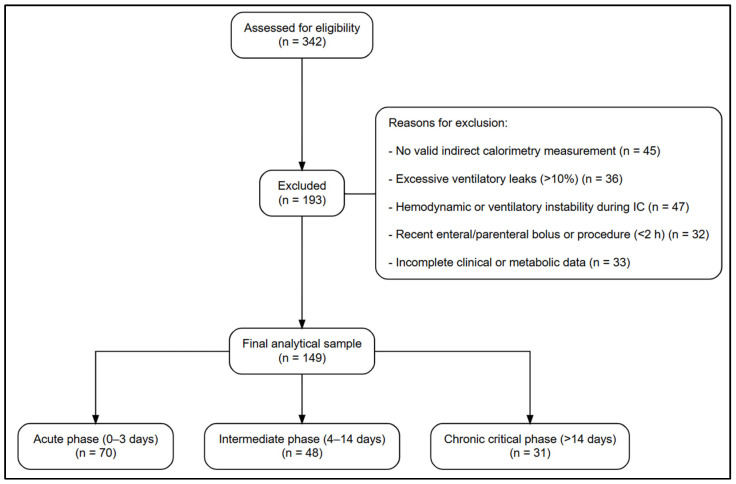
Flow diagram of patient selection and inclusion in the study. Mechanically ventilated adult patients assessed for eligibility in two tertiary-care intensive care units were retrospectively screened. Patients were excluded based on predefined clinical, ventilatory, and data-quality criteria. The final analytical cohort consisted of 149 patients, stratified according to the phase of critical illness at the time of indirect calorimetry assessment.

A total of 149 mechanically ventilated adults constituted the final analytical sample and were stratified by phase of critical illness: acute (0–3 days, n = 70), intermediate (4–14 days, n = 48), and chronic (>14 days, n = 31). Patients were recruited from two tertiary-care intensive care units. Details on patient screening, exclusions, and reasons for exclusion are presented in Figure 1.

Sex distribution was balanced across phases (*p* = 0.711), with males representing 56% of the cohort. The median age was 66 years (IQR 54–74) and did not differ significantly among phases (*p* = 0.912). Anthropometric characteristics—body weight (median 85 kg, IQR 75–98), height (median 1.67 m, IQR 1.60–1.75), ideal body weight (median 62.7 kg, IQR 57.2–67.6), and BMI (median 31.1 kg/m^2^, IQR 26–36)—were comparable across phases (all *p* > 0.15), reflecting a predominantly overweight and obese population.

Severity scores indicated moderate organ dysfunction across phases. Median APACHE II and SOFA scores were 15 (IQR 9–21) and 6 (IQR 2–9), respectively, with no statistically significant differences between groups (*p* = 0.858 and *p* = 0.119). The Charlson Comorbidity Index differed across phases (*p* = 0.005; ε^2^ = 0.058), with higher values observed in the acute phase compared with the intermediate (*p* adj = 0.0137) and chronic phases (*p* adj = 0.0403).

### 3.2. Phase-Dependent Variation in Metabolic and Bioenergetic Parameters

The distribution of metabolic states differed significantly across phases of critical illness (*p* = 0.002). Hypometabolic profiles were more frequent in the acute phase (64%) and less frequent in the intermediate (31%) and chronic phases (35%). In contrast, the proportion of hypermetabolic profiles increased from 20% in the acute phase to 44% and 48% in the intermediate and chronic phases, respectively (Table 1).

Resting energy expenditure differed across phases (*p* = 0.024, ε^2^ = 0.037), with median REE values increasing from 1664 kcal/day in the acute phase to 2074 kcal/day in the chronic phase. Post hoc analyses showed higher REE values in the chronic phase compared with the acute phase (*p* adj = 0.0397), whereas no significant difference was observed between the intermediate and chronic phases (*p* adj = 0.46). Normalized indices of energy expenditure also differed across phases, including REE/kg (*p* = 0.006) and REE/IBW (*p* = 0.024). REE/kg was higher in the intermediate phase than in the acute phase (*p* adj = 0.0040).

**Figure 2:**
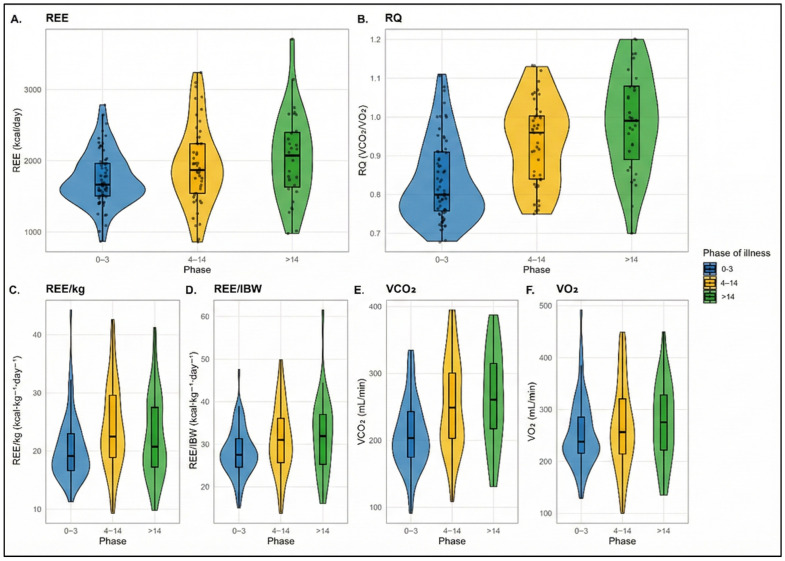
Comparative distribution of metabolic and gas-exchange parameters across phases of critical illness. (**A**) Resting energy expenditure (REE); (**B**) respiratory quotient (RQ); (**C**) REE normalized by actual body weight (REE/kg); (**D**) REE normalized by ideal body weight (REE/IBW); (**E**) carbon dioxide production (VCO_2_); and (**F**) oxygen consumption (VO_2_). Each violin plot depicts the probability density of values for each phase (0–3, 4–14, and > 14 days), with boxplots showing the interquartile range and median and dots representing individual measurements. Color coding denotes the phase of illness: blue for early (0–3 days), yellow for intermediate (4–14 days), and green for late (>14 days) phases.

The respiratory quotient varied markedly across phases (*p* < 0.001, ε^2^ = 0.240), increasing from a median of 0.80 in the acute phase to 0.99 in the chronic phase. Post hoc Dunn–Bonferroni comparisons indicated higher RQ values in both the intermediate and chronic phases compared with the acute phase (*p* adj < 0.0001), with no significant difference between the intermediate and chronic phases (*p* adj = 0.49) (Figure 2).

Carbon dioxide production differed significantly across phases (VCO_2_: *p* < 0.001, ε^2^ = 0.125), with higher values observed in both the intermediate (*p*_adj = 0.0013) and chronic phases (*p*_adj = 0.0005) relative to the acute phase. Body size–adjusted indices of VCO_2_ (VCO_2_/weight and VCO_2_/IBW) showed similar between-phase differences (both *p* < 0.001). Oxygen consumption did not differ significantly across phases (VO_2_: *p* = 0.313), and no statistically significant differences were observed for VO_2_ normalized by body weight or ideal body weight.

Overall, multiple metabolic and gas-exchange parameters exhibited statistically significant differences across phases of critical illness, with effect sizes ranging from small to large (ε^2^ or η^2^ ≈ 0.037–0.240), as summarized in Table 1.

### 3.3. Correlation Between REE and Clinical or Metabolic Parameters

Correlation analyses demonstrated strong associations between resting energy expenditure (REE) and gas-exchange variables. REE was strongly correlated with oxygen consumption (VO_2_; ρ = 0.956, 95% CI 0.939–0.968, *p* < 0.001) and carbon dioxide production (VCO_2_; ρ = 0.888, 95% CI 0.848–0.918, *p* < 0.001). In contrast, no statistically significant linear association was observed between REE and the respiratory quotient (RQ; ρ = 0.055, *p* = 0.517).

Regarding clinical parameters, REE was not significantly correlated with body mass index (BMI; ρ = 0.065, *p* = 0.433), APACHE II score (ρ = −0.144, *p* = 0.086), or SOFA score (ρ = −0.068, *p* = 0.416). Age showed a modest inverse correlation with REE (ρ = −0.281, 95% CI −0.423 to −0.126, *p* < 0.001).

Normalized indices were also examined. REE/kg and REE/IBW were correlated with absolute REE (ρ = 0.639 and 0.893, respectively; both *p* < 0.001). Weight-adjusted gas-exchange indices, including VO_2_/weight, VO_2_/IBW, VCO_2_/weight, and VCO_2_/IBW, showed moderate to strong correlations with REE (ρ range 0.533–0.846; all *p* < 0.001). Overall, correlation analyses identified consistent associations between REE and gas-exchange parameters, whereas correlations with anthropometric measures and severity scores were weak or non-significant.

### 3.4. Multivariable Modeling

Model 1: Determinants of REE (kcal/day)

A multivariable linear regression model was fitted to evaluate associations between clinical variables and measured resting energy expenditure (REE). The model included phase of illness, body mass index (BMI), SOFA score, Acute Physiology and Chronic Health Evaluation II (APACHE II) score, Charlson Comorbidity Index, and carbon dioxide production (VCO_2_).

After adjustment, VCO_2_ was positively associated with REE (β = 6.8, 95% CI 6.2–7.4; *p* < 0.001). BMI was also positively associated with REE (β = 8.0, 95% CI 3.1–13.0; *p* = 0.002). SOFA score showed an inverse association with REE (β = −14, 95% CI −26 to −2.4; *p* = 0.018). No statistically significant associations were observed for APACHE II score or the Charlson Comorbidity Index.

Phase of illness, modeled using orthogonal polynomial contrasts, showed a statistically significant linear component (phase.L β = −107, 95% CI −179 to −35; *p* = 0.004), whereas the quadratic component was not statistically significant (phase.Q β = 27, 95% CI −38 to 92; *p* = 0.40).

Model diagnostics indicated mild heteroscedasticity and minor deviations from normality in residual distributions. Heteroskedasticity-consistent (HC3) robust standard errors were therefore applied. No evidence of problematic multicollinearity or residual autocorrelation was observed. Results are summarized in Table 2.

Model 2: REE/IBW and Metabolic Phenotype

A second multivariable linear regression model was fitted using resting energy expenditure normalized by ideal body weight (REE/IBW, kcal·kg^−1^·day^−1^) as the dependent variable. Covariates included phase of illness, BMI, SOFA score, respiratory quotient (RQ), VCO_2_, and metabolic phenotype.

In this model, VCO_2_ was positively associated with REE/IBW (β = 0.07, 95% CI 0.06–0.08; *p* < 0.001). BMI was also positively associated with REE/IBW (β = 0.24, 95% CI 0.17–0.32; *p* < 0.001). RQ showed an inverse association with REE/IBW (β = −15, 95% CI −18 to −11; *p* < 0.001). Phase of illness and SOFA score were not significantly associated with REE/IBW.

Metabolic phenotype was associated with REE/IBW. Compared with the normometabolic reference category, hypometabolic status was associated with lower REE/IBW (β = −2.8 kcal·kg^−1^·day^−1^, 95% CI −4.1 to −1.5; *p* < 0.001), whereas hypermetabolic status was associated with higher REE/IBW (β = 3.2 kcal·kg^−1^·day^−1^, 95% CI 1.7–4.7; *p* < 0.001).

Model diagnostics indicated acceptable multicollinearity (VIF < 1.5), minimal heteroscedasticity (Breusch–Pagan *p* = 0.28), mild deviations in residual tails (White test *p* = 0.038), and no evidence of residual autocorrelation (Durbin–Watson = 1.96). Heteroskedasticity-consistent (HC3) robust standard errors were applied. The results are presented in Table 3.

### 3.5. Model Performance and Validation

Model performance metrics differed between the two analytical approaches. The model using absolute resting energy expenditure (Model 1: REE) achieved an adjusted R^2^ of 0.83, with root mean square error (RMSE) of 209.8 and mean absolute error (MAE) of 156.3. The model using resting energy expenditure normalized by ideal body weight (Model 2: REE/IBW) showed an adjusted R^2^ of 0.87, with lower RMSE (2.66) and MAE (1.92).

Information criteria favored the normalized model. Compared with Model 1, Model 2 yielded lower Akaike Information Criterion (AIC: 705.5 vs. 1952.8), corrected AIC (AICc: 707.2 vs. 1954.1), and Bayesian Information Criterion (BIC: 735.1 vs. 1979.4).

Ten-fold cross-validation results were consistent with the primary analyses. For Model 1, cross-validated RMSE and MAE were 220.6 and 170.9, respectively. For Model 2, cross-validated RMSE was 2.84 and MAE was 2.07.

Restricted cubic spline analyses did not improve model fit for absolute REE (AICc = 1954.1 for the linear specification vs. 1963.4 for spline-based models). For the REE/IBW model, spline-based specifications showed lower AICc values compared with the linear model (694.3 vs. 707.2), indicating modest nonlinearity in the relationship between predictors and normalized REE.

Because data were derived from two independent intensive care units, additional sensitivity analyses were performed to account for ICU site. Inclusion of ICU as an adjustment factor and stratified refitting of models by ICU did not materially alter model coefficients, goodness-of-fit indices, or prediction error metrics. These analyses are reported to contextualize model performance and validation across sites.

## 4. Discussion

In this multicenter retrospective study of 149 mechanically ventilated adults, we observed a phase-dependent pattern in energy metabolism across the course of critical illness, characterized by increases in resting energy expenditure from the acute to the chronic phase and parallel changes in weight-normalized indices (REE/kg and REE/IBW). In parallel, the distribution of metabolic phenotypes shifted, with hypometabolic profiles predominating in the early phase and hypermetabolic profiles becoming more frequent in later phases. These findings align with the concept of a complex and evolving energy expenditure response in critical illness, where indirect calorimetry reveals dynamic variation that predictive equations cannot fully capture [16].

Among gas-exchange parameters, the respiratory quotient (RQ) exhibited the largest phase-related variation, increasing progressively across illness stages. Importantly, despite these marked shifts, RQ was not associated with absolute REE, indicating that substrate utilization changes predominantly affect the composition, rather than the magnitude, of energy expenditure. Carbon dioxide production (VCO_2_) increased across phases and tracked changes in REE, whereas oxygen consumption (VO_2_) showed only a modest trend. In clinical practice, IC systems derive both REE and RQ from VO_2_ and VCO_2_ measurements using established formulas (e.g., Weir formula).

In multivariable analyses, VCO_2_ emerged as the strongest independent predictor of absolute REE, whereas illness-severity scores such as APACHE II and SOFA showed limited associations once gas-exchange variables were included. Body mass index retained a modest positive association with REE. These results are consistent with evidence that predictive equations frequently over- or underestimate energy expenditure in critically ill patients, and that methods incorporating dynamic respiratory data often perform better than static models [17,18].

When REE was normalized by ideal body weight, model parsimony and predictive performance improved, and metabolic phenotype, BMI, and RQ emerged as independent correlates in the normalized model [19,20]. Normalization likely reduces scale-dependent variability, making comparisons across heterogeneous ICU populations more interpretable—especially in cohorts with high prevalence of overweight and obesity. This analytical benefit has been noted in recent nutritional research emphasizing individualized assessment [13].

Our findings align with prospective and observational studies demonstrating that metabolic demands evolve substantially during critical illness and are frequently mischaracterized by predictive equations, particularly in mechanically ventilated patients [21]. Prior work in heterogeneous ICU cohorts, including ventilated COVID-19 populations, demonstrates progressive hypermetabolism that is inadequately captured by standard formulas, emphasizing the need for serial IC [22]. Mechanistically, these metabolic shifts reflect acute-phase inflammatory activation, mitochondrial dysfunction, catecholaminergic stimulation, and increasing exogenous carbohydrate delivery as nutrition advances through the ICU stay [23]. Although RQ evolved markedly across phases, this shift did not independently influence absolute REE in our adjusted models, indicating that substrate preference and total energy turnover are partially dissociable.

The systematic misestimation of REE by predictive equations has been associated with clinically relevant consequences, including energy deficits, excessive CO_2_ production, ventilatory load, and worse clinical outcomes [24]. Meta-analytic evidence indicates that IC-guided nutrition improves survival compared with equation-based strategies, particularly when energy delivery is matched to measured needs [25]. Consistent with this, our results reinforce the limited predictive value of severity scores (APACHE II, SOFA) for estimating energy requirements [26], whereas moment-to-moment metabolic flux—expressed predominantly through VCO_2_—remains the dominant determinant of energy expenditure [27]. In contrast, RQ reflected shifts in substrate use but did not independently predict REE in our cohort. These observations support the use of IC not only for caloric prescriptions, but also as a monitoring tool to align nutrition with the evolving metabolic phenotype of critical illness [20].

### 4.1. Mechanistic Insights

Inflammation, catecholamines, glucocorticoids, and insulin resistance drive accelerated gluconeogenesis and glycolysis, favoring carbohydrate oxidation and higher RQ; concurrently, lipid oxidation may be suppressed in later phases, especially under high carbohydrate delivery conditions [23]. Increased VCO_2_ reflects both metabolic CO_2_ generation and buffering of acid–base loads, with clinical impact on ventilator settings and work of breathing [28]. Device studies highlight that accurate VO_2_/VCO_2_ capture is essential—portable IC systems can overestimate VO_2_/VCO_2_, necessitating calibration and cross-device harmonization [29]. Emerging continuous IC sensors promise 24/7 trend monitoring that may enhance feed titration and ventilatory management [30]. Nevertheless, these mechanistic shifts did not, in isolation, alter absolute REE once VCO_2_ was incorporated into the statistical model.

### 4.2. Clinical Implications

1. Phase-adaptive energy prescriptions. Early hypometabolism warrants caution to avoid exogenous overfeeding, whereas chronic phases demand higher caloric targets to prevent cumulative deficits—best determined by serial IC rather than formulas [22,26,31].
2. Metabolic phenotyping to guide macronutrients. As RQ approaches 1.0, limit carbohydrate fraction; consider balanced protein provision and cautious lipids, titrating to gas-exchange to avoid excessive CO_2_ production that could prolong ventilation [24,32].
3. Ventilatory implications. Rising VCO_2_ implies potential increases in minute ventilation to maintain PaCO_2_; feed composition should be coordinated with respiratory therapy, particularly in patients with limited ventilatory reserve [20,33].
4. Operationalizing IC. Where a full IC is unavailable, validated surrogates (e.g., ultrasound models) may assist, though accuracy declines in early acute phases or low BMI—hence IC remains preferred [34].

The strengths of this study include its multicenter design, which enhances external validity, and the use of standardized indirect calorimetry procedures applying strict steady-state criteria to ensure high-quality metabolic measurements. The comprehensive characterization of energy metabolism—combining continuous parameters (REE, VO_2_, VCO_2_, RQ) with categorical metabolic phenotyping—allowed a nuanced assessment of metabolic dynamics across phases of critical illness. Furthermore, the analytical framework incorporated robust statistical procedures, including heteroskedasticity-consistent estimators, multicollinearity diagnostics, and cross-validation, providing methodological rigor and supporting the internal validity of the findings.

Nonetheless, several limitations deserve careful consideration. The retrospective design of the study and the reliance on a single calorimetry measurement per patient limit the ability to analyze intra-individual metabolic trajectories over time, which are very relevant in critical illness. The lack of direct body composition measurements, such as CT-based muscle cross-sectional area or bioimpedance analysis, hinders interpretation of REE in relation to lean mass depletion, an important factor in metabolic efficiency. Additionally, using different IC devices across centers introduces the potential for inter-device variability, highlighting the need for standardized calibration and harmonized measurement protocols in future research. Furthermore, since RQ was not independently linked to absolute REE, future studies should explore whether high-frequency metabolic monitoring more effectively captures the physiological relationships among substrate use, energy turnover, and clinical outcomes.

Looking forward, prospective research should evaluate whether phase-adaptive energy prescriptions guided by serial indirect calorimetry—preferably with continuous or high-frequency monitoring—improve clinically relevant outcomes such as duration of mechanical ventilation, muscle mass preservation, and functional recovery compared with equation-based strategies. Emerging ventilator-agnostic calorimetry technologies may facilitate real-time titration of energy delivery and ventilatory settings, although their clinical performance requires rigorous validation [30]. Integrative approaches that combine indirect calorimetry with body-composition metrics and biomarkers of mitochondrial function may further refine precision nutrition strategies and help mitigate muscle catabolism during prolonged critical illness.

## 5. Conclusions

This study shows that resting energy expenditure in mechanically ventilated critically ill adults was more strongly associated with gas-exchange variables than with anthropometric characteristics or illness-severity indices. Carbon dioxide production (VCO_2_) was independently associated with absolute REE, whereas oxygen consumption (VO_2_), despite its strong correlation with REE, did not retain independent significance after multivariable adjustment. Although respiratory quotient varied markedly across illness phases, it was not independently associated with absolute REE, suggesting that changes in substrate utilization primarily affect the composition rather than the magnitude of energy expenditure.

Normalization of REE by ideal body weight (REE/IBW) was associated with improved model performance, including reduced heteroscedasticity and greater parsimony. Within this normalized framework, metabolic phenotype, body mass index, and respiratory quotient were independently associated with REE/IBW, indicating that substrate utilization patterns and body-size–adjusted metabolic characteristics contribute to variability in normalized energy expenditure beyond illness severity or phase alone.

Overall, the combined use of indirect calorimetry and multivariable modeling provides a methodologically robust approach for characterizing metabolic patterns in critical illness. The present findings suggest that gas-exchange dynamics and metabolic phenotype may offer complementary information to traditional severity scores when assessing energy requirements. Future prospective studies are needed to determine whether integrating these parameters into nutrition-support strategies improves clinical outcomes.

## Figures and Tables

**Table 1:** Comparison of clinical and metabolic variables across critical illness phases.

Variable	0–3 Days (N = 70) ^1^	4–14 Days (N = 48) ^1^	>14 Days (N = 31) ^1^	Overall (N = 149)	*p*-Value ^2^	Effect Size ^3^
**Sex (male)**					0.711	— ^4^
Female	30 (43%)	23 (48%)	12 (39%)	65 (44%)		
Male	40 (57%)	25 (52%)	19 (61%)	84 (56%)		
**Metabolic state**					0.002	—
Hypometabolic	45 (64%)	15 (31%)	11 (35%)	71 (48%)		
Normometabolic	11 (16%)	12 (25%)	5 (16%)	28 (19%)		
Hypermetabolic	14 (20%)	21 (44%)	15 (48%)	50 (34%)		
**Age (years)**	66 (55, 74)	68 (51, 77)	66 (56, 73)	66 (54, 74)	0.912	ε^2^ ≈ 0.000
**Weight (kg)**	87 (75, 100)	81 (70, 93)	85 (80, 100)	85 (75, 98)	0.163	ε^2^ = 0.011
**Height (m)**	1.65 (1.60, 1.74)	1.67 (1.60, 1.75)	1.70 (1.60, 1.77)	1.67 (1.60, 1.75)	0.353	η^2^ = 0.014
**Ideal body weight (kg)**	62.7 (57.2, 67.0)	61.7 (57.2, 67.9)	65.4 (58.7, 69.2)	62.7 (57.2, 67.6)	0.396	η^2^ = 0.013
**BMI (kg/m^2^)**	32 (28, 35)	29 (25, 35)	32 (26, 39)	31 (26, 36)	0.238	ε^2^ = 0.006
**APACHE II**	15 (9, 21)	16 (11, 21)	14 (9, 20)	15 (9, 21)	0.858	ε^2^ ≈ 0.000
**SOFA at measurement**	5.0 (1.0, 8.0)	6.0 (4.0, 9.0)	6.0 (3.0, 9.0)	6.0 (2.0, 8.5)	0.119	ε^2^ = 0.016
**Charlson Comorbidity Index**	4.00 (2.00, 6.00)	2.00 (0.00, 4.00)	2.00 (1.00, 4.00)	3.00 (1.00, 5.00)	0.005	ε^2^ = 0.058
**REE (kcal/day)**	1664 (1505, 1964)	1869 (1539, 2239)	2074 (1603, 2412)	1781 (1523, 2177)	0.024	ε^2^ = 0.037
**REE/kg (kcal·kg^−1^·day^−1^)**	19 (17, 23)	22 (19, 30)	21 (17, 28)	21 (17, 26)	0.006	ε^2^ = 0.057
**REE/IBW (kcal·kg^−1^·day^−1^)**	28 (25, 31)	31 (26, 36)	32 (25, 38)	29 (25, 34)	0.024	ε^2^ = 0.037
**RQ (VCO_2_/VO_2_)**	0.80 (0.76, 0.91)	0.96 (0.84, 1.01)	0.99 (0.87, 1.08)	0.89 (0.79, 1.00)	<0.001	ε^2^ = 0.240
**VO_2_ (mL/min)**	238 (215, 287)	257 (213, 322)	276 (222, 330)	250 (217, 310)	0.313	ε^2^ = 0.002
**VCO_2_ (mL/min)**	204 (175, 243)	249 (200, 301)	261 (217, 315)	218 (188, 282)	<0.001	ε^2^ = 0.125
**VO_2_/weight (mL·min^−1^·kg^−1^)**	2.79 (2.44, 3.28)	3.11 (2.59, 4.05)	2.79 (2.33, 3.78)	2.85 (2.47, 3.65)	0.066	ε^2^ = 0.024
**VO_2_/IBW (mL·min^−1^·kg^−1^)**	3.95 (3.48, 4.63)	4.29 (3.50, 5.07)	4.28 (3.46, 5.09)	4.03 (3.48, 4.90)	0.357	ε^2^ ≈ 0.000
**VCO_2_/weight (mL·min^−1^·kg^−1^)**	2.29 (1.87, 2.68)	3.25 (2.49, 3.89)	2.72 (2.26, 3.77)	2.57 (2.08, 3.44)	<0.001	ε^2^ = 0.132
**VCO_2_/IBW (mL·min^−1^·kg^−1^)**	3.26 (2.85, 3.81)	4.00 (3.50, 4.86)	4.42 (3.28, 4.73)	3.60 (3.01, 4.46)	<0.001	ε^2^ = 0.148

^1^ Data expressed as n (%) for categorical variables and as median (Q1, Q3) for continuous variables. ^2^ *p*-values obtained using the Pearson χ^2^ test for categorical variables, the Kruskal–Wallis test for non-normal continuous variables, or one-way ANOVA when assumptions of normality and homoscedasticity were met (Shapiro–Wilk and Levene tests). ^3^ Effect size reported as η^2^ for ANOVA and ε^2^ for Kruskal–Wallis; interpreted as small (< 0.06), moderate (0.06–0.14), or large (>0.14). ^4^ Em dashes (—) indicate that effect size estimates were not applicable or not calculated for categorical variables.

**Table 2:** Multivariable linear regression model for determinants of REE (robust HC3 SE).

Predictor	β	95% CI	*p*-Value
(Intercept)	54	−163, 271	0.60
Phase of illness			
• Linear component (fase.L)	−107	−179, −35	0.004
• Quadratic component (fase.Q)	27	−38, 92	0.40
Body mass index (BMI, kg/m^2^)	8.0	3.1, 13	0.002
SOFA score	−14	−26, −2.4	0.018
APACHE II score	1.1	−4.3, 6.5	0.70
Charlson Comorbidity Index	−4.5	−19, 10	0.50
Carbon dioxide production (VCO_2_, mL/min)	6.8	6.2, 7.4	<0.001

Abbreviation: CI = confidence interval. • indicates polynomial terms for the phase of illness (linear and quadratic components).

**Table 3:** Multivariable linear regression model for REE/IBW and metabolic phenotype (robust HC3 SE).

Predictor	β	95% CI	*p*-Value
(Intercept)	19	15, 24	<0.001
Phase of illness			
• Linear component (fase.L)	−0.23	−1.2, 0.72	0.60
• Quadratic component (fase.Q)	−0.74	−1.6, 0.10	0.084
Body mass index (BMI, kg/m^2^)	0.24	0.17, 0.32	<0.001
SOFA score	0.00	−0.12, 0.13	>0.90
Respiratory quotient (RQ)	−15	−18, −11	<0.001
Carbon dioxide production (VCO_2_, mL/min)	0.07	0.06, 0.08	<0.001
Metabolic phenotype			
• Normometabolic (reference)	—	—	—
• Hypometabolic	−2.8	−4.1, −1.5	<0.001
• Hypermetabolic	3.2	1.7, 4.7	<0.001

Abbreviation: CI = confidence interval. • indicates polynomial terms for the phase of illness (linear and quadratic components). — indicates the reference category.

## Data Availability

Data supporting the findings of this study are available from the corresponding author upon reasonable request. Due to clinical privacy regulations, raw indirect calorimetry files cannot be publicly released.
